# As naturalistic as it gets: subtitles in the English classroom in Norway

**DOI:** 10.3389/fpsyg.2014.01510

**Published:** 2015-01-09

**Authors:** Mila Vulchanova, Lisa M. G. Aurstad, Ingrid E. N. Kvitnes, Hendrik Eshuis

**Affiliations:** Language Acquisition and Language Processing Lab, Department of Language and Literature, Norwegian University of Science and Technology (NTNU)Trondheim, Norway

**Keywords:** second language acquisition, subtitles, authentic target language input, comprehension, vocabulary

## Abstract

This study aimed to investigate the effects of subtitles in the context of authentic material on second language comprehension and potentially, second language acquisition for Norwegian learners of English. Participants in the study were 49 17-year-old students and 65 16-year-old students, who were all native speakers of Norwegian learning English as an L2 in high school. Both age groups were divided into three Conditions, where one group watched an episode of the American animated cartoon Family Guy with Norwegian subtitles, one group with English subtitles, and one group watched the episode with no subtitles. On a comprehension questionnaire conducted immediately after watching the episode positive short-term effects of both native language (L1) and target language (L2) subtitles were found for both age groups. However, no differences in terms of the language of the subtitles were found in the older and more advanced group. Four weeks later the participants responded to a word definition task and a word recall task to investigate potential long-term effects of the subtitles. The only long-term effect was found in the word definition task and was modulated by age. We found, however, that native language subtitles impact negatively on performance on the comprehension task. The results from this study suggest that the mere presence of subtitles as an additional source of information enhances learners' comprehension of the plot and content in animated audio-visual material in their L2. The absence of differences in terms of the language of the subtitles in the more advanced group suggests that both intralanguage and interlanguage subtitles can aid target language comprehension in very advanced learners, most probably due to better consolidated vocabulary knowledge in that group. The two groups differed also on predictors of performance on the two lexical tasks. While in the less proficient younger group, vocabulary status best predicted performance on both tasks (vocabulary predicts vocabulary), for the very advanced older group, grammar was a stronger predictor, highlighting the importance of generic language competence and skills in L2 tasks for highly proficient L2 users. We also found an effect of written L2 skills on performance on both lexical tasks indicative of the role of orthography in vocabulary consolidation.

## Introduction

In the Norwegian context, second language learning typically takes place in schools. However, learners are often exposed to input from the target language outside the school setting through television, movies, newspapers and the internet (Dahl and Vulchanova, 2014). Audio-visual material is a frequently used resource for teaching and learning English as an L2 and it provides learners with natural spoken dialog in the target language. Audio-visual material can be presented to learners without any subtitles, with native language (L1) subtitles, or with target language (L2) subtitles. From the point of view of SLA in a naturalistic environment, the question is what type of subtitles, if any, impact on learners' comprehension. The current study aimed to address the role of subtitled audio-visual material in L2 comprehension in the Norwegian context.

Linguistic input in the target language is essential for second language acquisition (Verspoor et al., 2009; Ellis, 2013), and exposure to such input can be obtained through reading and listening. When assessing the extent to which learners can benefit from exposure to L2 input one needs to take into account the learners' outset in terms of level of proficiency. There seems to be a consensus that an incipient learner cannot benefit from the same amount of input to the same extent as a more proficient learner due to processing limitations (Chiquito, 1995; Verspoor et al., 2009). Gilmore (2007) nevertheless argues that authentic input should not be adapted to the proficiency level of the L2 learner when exposing SLA learners to input in the target language. In this way, the target language is presented in a naturalistic manner, thus offering a far richer sample of the target language than adjusted materials (Gilmore, 2007; Benavent and Peñamaría, 2011). Authentic material, thus, provides learners with natural samples of the target language, facilitating the SLA process by advancing linguistic competence in the target language (Verspoor and Winitz, 1997). Audio-visual material can be a good source of authentic input, as suggested by Gilmore (2007), and even though authentic input can be argued to be too challenging for L2 learners (Day, 2003; Flowerdew and Peacock, 2011), the use of animated cartoons can be argued to provide authentic input in the classroom. Cartoons often involve more clearly enunciated speech in standard accents in the target language (Sherman, 2003). The bright colors and exaggerated intonation and other features can also increase the motivation of L2 learners, thus creating a better environment for learning (Sherman, 2003; Bahrani and Soltani, 2011). Bahrani and Soltani (2011) further recommended the use of animated cartoons in classroom activities, as they provide variation for the brain in engaging both the left and right hemisphere, and prevent the students from being bored.

Audio-visual material can be a particularly good source for authentic input, when accompanied by subtitles (Neuman and Koskinen, 1992; Baltova, 1999; Bianchi and Ciabattoni, 2008). The combination of auditory input in the L2, nonverbal visual information, and verbal visual (orthographic) information can be argued to contribute to a better SLA learning environment than when only two or only one of the three information channels are available (Baltova, 1999). The textual information serves as an extra source of linguistic input either in the L1 or the L2. In a number of eye-tracking studies, d'Ydewalle and Van de Poel (1999) and Danan (2004) note that readers automatically read the subtitles, whenever they are available, indicating that the auditory and the verbal textual information are processed in parallel. These studies suggest that subtitles do not necessarily hinder the processing of the auditory information (d'Ydewalle and Gielen, 1992). Furthermore, the eye-tracking study by Bisson et al. (2014) suggests that participants spend time attending to both the subtitles and the visual images, thus making use of both channels. Whether subtitles in the L1 (also called interlingual) or in the L2 (intralingual subtitles) in an auditory context are more facilitative has been debated, with experiments showing controversial results depending on the aspect of language being tested and the age and level of proficiency of the participants. A number of researchers (Vanderplank, 1988; Markham, 1999; Bird and Williams, 2002; Danan, 2004; Mitterer and McQueen, 2009; Gunderson et al., 2011; Vandergift, 2011; and Bianchi and Ciabattoni, 2008) have found that L2 subtitles are more facilitatory. It can be argued that intralingual (target language) subtitles are useful, since they allow the learner to map phonology directly onto orthographic representations, and thus enhance speech segmentation making processing and comprehension of the auditory material much easier (Bird and Williams, 2002; Mitterer and McQueen, 2009). Other results, however, (cf. Guillroy, 1998; d'Ydewalle and Van de Poel, 1999; Bianchi and Ciabattoni, 2008; Zarei and Rashvand, 2011) suggest that L1 subtitles are more facilitatory. The results of Bianchi and Ciabattoni (2008) are particularly interesting for the current study, as they found that L1 subtitles were more facilitatory for the less proficient learners, whereas L2 subtitles were more facilitatory for the more advanced learners in their study. They argue that this difference might be due to L1 subtitles being automatically processed, while L2 subtitles may require more advanced knowledge of the L2, in order to have a positive effect (Guillroy, 1998; Bianchi and Ciabattoni, 2008). Guillroy (1998) also argues that L2 subtitles cannot compensate for difficult vocabulary and fast speech in audio-visual material. Therefore, the long term effects of target language subtitles, and subtitles in general, is still unclear (Vandergift, 2011).

### Hypotheses

In this study, we hypothesized that subtitles would enhance participants' performance both on comprehension and on vocabulary learning (word definition and word identification), thus indicating facilitatory short-term and long term effects of subtitles in language learning. If the subtitles were indeed found to have an effect, we further hypothesized that the younger and less advanced group (16-year-old students) would benefit more from the Norwegian (L1) subtitles, while the older and more advanced group (17-year-old students) would benefit more from the English (L2) subtitles.

## Materials and methods

### Participants

Two age-groups were recruited for the study, a group of 16-year-old students (*N* = 65; *F* = 34, *M* = 31) and a group of 17-year-old students (*N* = 49; *F* = 24, *M* = 25). All participants were monolingual native speakers of Norwegian and attended the same school in a big city in Norway. The 17-year-old group was assumed to have a higher level of proficiency in English, as this was a more homogenous group of students who had all chosen to study English as one of their high school specialization subjects, whereas for the younger group English was still a compulsory school subject. The study was conducted in the participants' high school classrooms at times when they normally received instruction in English, making the environment for the research more natural.

The participants were divided into three Conditions (sub-groups) for each age group using their original school class classification. This grouping, based on original class classification led to the experiments being conducted during different times of the day based on the school schedule, and also caused the gender distribution to vary somewhat between the different sub-groups. All the students in all the six original classes were encouraged to participate in the study. Prior to the study, informed parental consent was elicited from the parents of all participants allowing the students to participate in the study, since the students were all under the age of majority. The study was also approved formally by the school and the teachers. Participants from both age groups were divided into three conditions, where one group watched the episode with Norwegian subtitles (Norwegian subtitles group), one with English subtitles (English subtitles group), and one with no subtitles (Control group).

The particular purpose of the study was not revealed to either students, teachers, or parents; they were only informed that the study investigated second language acquisition. Students not willing to participate or absent on one of the testing days, were not included in the study and the analyses. After all the testing had been completed, some further participants were excluded due to missing background information from the background questionnaire, and were dropped from further analyses of the results. Also participants who reported severe hearing or visual problems or other language related problems that might have had an effect on the results were excluded. Participants with an L1 that was not Norwegian were also excluded. All participant information was treated anonymously and according to the rules prescribed by the Norwegian Data Protection Board which approved the study.

### Materials and procedure

In order to establish a baseline, prior to the study, all participants were tested in English grammar and vocabulary competence. The grammar test was the Cambridge Essential Grammar in Use Level Test (available at http://www.cambridge.org/other_files/Flash_apps/inuse/EssGramTest/EssGramIndex.htm). In this test the participant fills in a blank choosing the correct option from among four alternatives. The test comprises 50 sentences and each item targets key areas of grammar (word forms, verbs, and verb forms, parts of speech, adverbials, word order). The maximum score is thus 50. The vocabulary test estimates participants' vocabulary size on the basis of performance on 10 word definitions (http://dynamo.dictionary.com/placement/level). This test has 4 levels, elementary school level, middle school level, high school level and college and beyond level. The degree of difficulty changes for each next level. The test is designed for native speakers of English. A typical score at level “College and beyond” can range between 45 and 55,000 words. Participants took the Word Dynamo test twice (in October 2012) responding at level “Middle school,” and an average score was, thus, calculated for each participant. Both tests were conducted on-line on the internet, and the scores from these two tests were recorded by an experimenter. In addition, participants completed a background questionnaire (Appendix 4 in Supplementary Material) requesting information about their linguistic background, and focusing on extra-curricular activities where English as a second language might be involved, thus offering information on factors (variables) which impact on the process of second language acquisition, and as such, might potentially influence performance on the tasks. This questionnaire provided information on self-assessed L2 skills (speaking, reading, writing, and listening) on a scale from “basic” (1) to “fluent” (4); frequency of reading and writing English; frequency of watching English films and cartoons; choice of subtitle viewing when watching English movies (English/Norwegian/no); frequency of playing computer games in English and whether they had watched the *Family Guy* series (an episode of which was the experimental video). Frequency responses were on a scale from “rarely” (1) to “every day” (5). For the purposes of the analyses, only the numerical values of these responses, and their means, were used. The background questionnaire, the comprehension questionnaire, and the word recall task were all in paper format, and the participants responded using a pen/pencil.

When all participants had responded to the grammar and vocabulary tests and the background questionnaire, they watched an episode of the American animated cartoon Family Guy. The episode lasted approximately 20 min and was only watched once. The dialog was in standard American and British accents and was believed to be comprehensible to the participants, and the plot was presumed to be fairly easy to follow. We decided to use an animated cartoon episode, as cartoons often include more enunciated speech, and are believed to be more easily understood by the audience (Sherman, 2003). Moreover, a cartoon was expected to motivate the participants into paying more attention, thus creating a reliable context for the study (Sherman, 2003; Bahrani and Soltani, 2011).

In order to investigate the potential short term effects of subtitles on the comprehension of the episode, the participants responded to a comprehension questionnaire in a multiple choice format immediately after watching the episode (Appendix 1 in Supplementary Material). Four weeks later the participants responded to a word definition task in a multiple choice format (Appendix 2 in Supplementary Material) and a word recall task (Appendix 3 in Supplementary Material), both including words and phrases the participants had encountered in the Family Guy episode they had watched previously. This was done in order to investigate potential long term effects of the subtitles. The word definition task was administered in a multiple choice format and consisted of 30 words and phrases that occurred in the episode. In this task, participants were asked to select the correct definition from four alternatives. The word recall task consisted of a list of 53 words of which 22 occurred in the episode and 31 did not occur in the episode (which acted as distractors). The 22 target items selected for the word recall task were all semantically related to the plot of the episode. Thus, our expectation was that there was a likelihood that participants would recognize them, if they had processed the episode at a deeper (semantic) level. Participants were asked to identify the words they believed had occurred in the episode. The frequency of the words in both the word definition task and the word recall task was established in the Corpus of Contemporary American English (COCA) and was included as a potential factor in the inferential analysis. For all three tasks, instructions were written on top of the paper as well as given orally by the experimenters.

## Analyses and results

Inferential statistics was conducted in R using a generalized linear mixed model fit by the Laplace approximation to check for dependencies between the results from the tests, the presence/absence and nature of subtitled stimuli and variables from the participants' background (Baayen et al., 2008; Bolker et al., 2009). The models in R were created by using the factors from the initial background testing and the vocabulary and grammar test results. The results from the comprehension questionnaire, the word definition task, and the word recall task were analyzed independently (coded as correct or incorrect response for each item). Age and subtitle condition were included as between subject factors, test item and subject were included as random effects, while word frequency and the results of the grammar test, the vocabulary test and the background questionnaire were included as covariates. The models created in R were compared using likelihood ratio tests (ANOVA) in order to find the best fitting models. The best fitting models for each of the tasks are presented in the Results Section below. The ratio between the different groups varied from test to test, and the models will therefore have different variables as predictors of the results.

### Results

#### Initial L2 proficiency results

The average scores on the grammar and vocabulary tests are presented in Tables 1, 2. On the grammar test, the scores were calculated as the number of correct responses out of 50, and on the vocabulary test the scores were calculated as the estimated amount of English words known to the participants (see http://dynamo.dictionary.com/placement/level).

**Table 1:** **Average score on pre-study grammar (Cambridge Essential Grammar in Use Test) and vocabulary tests (Word Dynamo) (17-year-old students)**.

**Condition (group)**	***N***	**Vocabulary**	**Grammar**
Norwegian subtitles	14	14 747.79	45.50
English subtitles	16	15 499.31	46.13
Control group	19	16 917.53	45.21

**Table 2:** **Average score on pre-study grammar (Cambridge Essential Grammar Test) and vocabulary tests (Word Dynamo) (16-year-old students)**.

**Condition (group)**	***N***	**Vocabulary**	**Grammar**
Norwegian subtitles	16	14 842.91	44.88
English subtitles	25	11 658.72	42.68
Control group	24	11 015.65	44.67

Tables 1, 2 show that the expected difference in L2 proficiency between the 16-year-old group and the 17-year-old group was justified. Overall, the 16-year-old students on average achieved lower scores on both the grammar and the vocabulary tests [ANOVA: *F*_(1, 108)_ = 7.467, *p* < 0.01 resp. *F*_(1, 108)_ = 20.235, *p* < 0.001]. For the vocabulary test also a significant interaction between Age and Subtitle Condition was found [*F*_(2, 108)_ = 5.755, *p* < 0.005]. Separate analyses for the Age groups revealed no significant differences for the 17-year-olds. For the 16-year-olds, participants in the Norwegian subtitles Condition significantly outperformed the Control and the English subtitles groups on the vocabulary test (Bonferroni corrected: *p* < 0.01 resp. *p* < 0.05). The high score of participants in this condition will be considered in more detail later.

#### Comprehension questionnaire

Positive short term effects of the subtitles were found for both the 16-year-olds and the 17-year-olds in the analysis of the comprehension questionnaire. The results from the comprehension questionnaire are shown in Tables 3–5 below (for each analysis the best-fitting model is shown; the number of included factors accordingly varies).

**Table 3:** **Results of the inferential analysis of the comprehension questionnaire (both age groups)**.

	**Estimate**	***SE***	***z*-value**	**Pr(>|z|)**
(Intercept)	−28.37734	5.71280	−4.967	6.79e-07^***^
Age	0.41712	0.24820	1.681	0.092854(^*^)
GroupEng	1.15136	0.26492	4.346	1.39e-05^***^
GroupNor	1.09320	0.29713	3.679	0.000234^***^
log(Voc)	1.19687	0.39036	3.066	0.002169^**^
log(Grammar)	2.95033	1.38667	2.128	0.033367^*^
FilmSubeng	0.60008	0.47325	1.268	0.204803
FilmSubnorw	−0.62278	0.24509	−2.541	0.011054^*^
Cartoon	−0.18873	0.08522	−2.215	0.026781^*^
Eng_game	0.40576	0.08350	4.859	1.18e-06^***^
Film_EngT	−0.32238	0.15565	−2.071	0.038346^*^
Read_Norw	0.29916	0.16017	1.868	0.061804(^*^)
Listen	0.91757	0.37791	2.428	0.015183^*^

Age, Age group; GroupEng, English subtitles Condition; GroupNor, Norwegian subtitles Condition; log(voc), vocabulary test results (log); log(Grammar), grammar test results (log); FilmSubeng, English subtitles when watching an English movie; FilmSubnor, Norwegian subtitles when watching an English movie; Cartoon, amount of time spent watching animated cartoons in English; Eng_Game, amount of time spent playing English computer games; Film_EngT, frequency of watching English films and series; Read_Norw, self-estimated Norwegian reading skills; Listen, composite factor created by the ratio of self-estimated English listening skills compared to self-estimated English speaking skills. Significance codes: (^*^) p ≤ 0.1; ^*^ p ≤ 0.05; ^**^ p ≤ 0.01; ^***^ p ≤ 0.001.

**Table 4:** **Results of the inferential analysis of the comprehension questionnaire (17-year-old group)**.

	**Estimate**	***SE***	***z*-value**	**Pr(>|z|)**
(Intercept)	−35.6168	13.9389	−2.555	0.01061^*^
GroupEng	1.5088	0.4979	3.030	0.00244^**^
GroupNor	1.5172	0.5004	3.032	0.00243^**^
EngGame	2.3338	0.5305	4.399	1.09e-05^***^
log(Grammar)	9.3537	3.6623	2.554	0.01065^*^
FGyes	0.6939	0.4045	1.716	0.08623 (^*^)

GroupEng, English subtitles Condition; GroupNor, Norwegian subtitles Condition; EngGame, composite factor created by the ratio of the amount of time spent playing English computer games compared to self-estimated English writing skills; log(Grammar), grammar test results (log); FGyes, have watched Family Guy before. Significance codes: (^*^) p ≤ 0.1; ^*^ p ≤ 0.05; ^**^ p ≤ 0.01; ^***^ p ≤ 0.001.

**Table 5:** **Results from analysis of comprehension questionnaire (16-year-old group)**.

	**Estimate**	***SE***	***z*-value**	**Pr(>|z|)**
(Intercept)	−12.74289	3.50561	3.635	0.000278^***^
GroupEng	0.78831	0.29695	2.655	0.007938^**^
GroupNor	0.75125	0.36342	2.067	0.038722^*^
log(voc)	1.33337	0.38792	3.437	0.000588^***^
listen_eng	0.65233	0.20861	3.127	0.001766^**^
Eng_game	0.18479	0.09895	1.868	0.061831 (*)

GroupEng, English subtitles Condition; GroupNor, Norwegian subtitles Condition; listen_eng, self-estimated English listening skills; log(voc), vocabulary test results (log); Eng_game, amount of time spent playing English computer games. Significance codes: (^*^) p ≤ 0.1; ^*^ p ≤ 0.05; ^**^ p ≤ 0.01; ^***^ p ≤ 0.001.

As can be seen in Table 3, there is only marginally a difference between the 16 and 17-year-olds with the older group giving more correct responses (level of significance *p* < 0.1). There are clear short term effects of the subtitles (*p* < 0.001). The availability of subtitles for participants in both the English and the Norwegian subtitles Conditions enhanced these participants' performance, indicating positive short term effects of the subtitles on comprehension of the contents of the episode. Interestingly, however, the analysis indicates that the language of the subtitles did not matter, as the effect of subtitles in both conditions is highly significant. This suggests that both intralingual and interlingual subtitles as a source of input facilitated the comprehension of the episode, with these two groups performing significantly better than the control group. The most significant factor, however, was the computer game factor, estimated as the amount of time the participants had spent playing English computer games (*p* < 0.001). Also, the results from the grammar test were significant (*p* < 0.05), suggesting that higher grammar competence, as indicated by better performance on the grammar test, was a reliable predictor of comprehension of the Family Guy episode. Likewise, the score on the vocabulary test (*p* < 0.01) significantly predicted performance on the comprehension task. Interestingly, having—in daily life—the habit of displaying Norwegian subtitles when watching English films and series is associated with lower scores on the task (*p* < 0.05), while displaying English subtitles increases performance (n.s.) as compared to not displaying subtitles at all. On the other hand, the amount of time that participants usually spend on watching animated cartoons in English (*p* < 0.05) decreases scores as does the more frequent watching of English films and series (*p* < 0.05). The composite factor created by the ratio of self-estimated English listening skills compared to self-estimated English speaking skills is tied to increased scores (*p* < 0.05). Finally, the self-estimated Norwegian reading skill is only marginally predicting results (*p* < 0.1).

The analysis of the results from the comprehension questionnaire for the 17-year-old group only indicates clear short term effects of the subtitles (level of significance *p* < 0.01). The availability of subtitles for participants in both the English and the Norwegian subtitles Conditions enhanced these participants' performance, indicating positive short term effects of the subtitles on comprehension of the contents of the episode. For that specific age-group, too, the analysis indicates that the language of the subtitles did not matter, as the effect of subtitles in both conditions is equally significant (almost identical *p*-values for the two Conditions/groups). The most significant factor in that age-group, however, was the composite computer game factor, estimated as the amount of time the participants had spent playing English computer games, compared to how proficient they estimated themselves to be at writing English from the L2 skill self-assessment part of the background questionnaire (*p* < 0.001). Also, the results from the grammar test were significant (*p* < 0.05), suggesting that higher grammar competence, as indicated by better performance on the grammar test, was a reliable predictor of comprehension of the Family Guy episode. The amount of time the participants had spent watching Family Guy before was also marginally significant (*p* < 0.1).

For the 16-year-old age group, participants in the English subtitles Condition performed significantly better than the two other groups (*p* < 0.01). Norwegian subtitles also seemed to enhance comprehension of the Family Guy episode (*p* < 0.05) for participants in that condition, however, this result should be seen in relation to this group's performance on the vocabulary test. The most significant predictor of comprehension in the 16-year-old group as a whole was the score on the vocabulary test (*p* < 0.001), something which might explain why the Norwegian subtitles were less significant than the English subtitles, since the Norwegian subtitles group performed better on the vocabulary task. Self-estimated English listening skills were also an important predictor (*p* < 0.01), suggesting that participants who were good at processing English aural material, were also good at understanding the video, and most likely used both the subtitles and the audio to process the contents. This factor also emerged as significant in the combined group analysis. In addition, the amount of time spent playing English computer games marginally predicted the results (*p* < 0.1).

#### Word definition task

We ran a combined analysis including Age (age group: 16|17) as an interacting factor. The results of the analysis are presented in Table 6 below. A fixed main effect was found for Grammar, English writing skills, and video viewing Condition, with native language subtitles facilitating performance on the task, but this effect was modulated by age. In addition, there was a negative effect of the habit of watching films with native language subtitles (as reported in the questionnaire). No effects of the subtitles were, however, found on the word definition task in separate age group analyses, indicating that there were no long term effects for each group independently of the type of subtitles included in the different experimental conditions. Other factors were, however, found to influence the participants' performance. The results from the inferential analysis are shown in Tables 7, 8.

**Table 6:** **Results of the inferential analysis of the word definition task (both age groups)**.

	**Estimate**	***SE***	***z*-value**	**Pr(>|z|)**
(Intercept)	−19.230810	8.334339	−2.307	0.02103^*^
Age	0.588566	0.445330	1.322	0.18629
GroupEng	3.727448	5.028121	0.741	0.45850
GroupNor	10.754150	5.256792	2.046	0.04078^*^
Sexmale	0.266994	0.142786	1.870	0.06150(^*^)
log(FreqWD)	−0.413048	0.215268	−1.919	0.05502(^*^)
log(Grammar)	2.877757	0.954838	3.014	0.00258^**^
FilmSubeng	−0.202369	0.236279	−0.856	0.39173
FilmSubnor	−0.341372	0.146421	−2.331	0.01973^*^
Cartoon	−0.090802	0.052144	−1.741	0.08162(^*^)
Read_Norw	−0.002307	0.094375	−0.024	0.98050
Read_Eng	0.199213	0.129938	1.533	0.12524
Write_Eng	0.392285	0.126716	3.096	0.00196^**^
Listen_Eng	0.233386	0.131074	1.781	0.07498(^*^)
Total_Eng	−0.300474	0.184938	−1.625	0.10422
Age:GroupEng	−0.224494	0.306690	−0.732	0.46418
Age:GroupNor	−0.630797	0.319647	−1.973	0.04845^*^

Age, Age group; GroupEng, English subtitles Condition; GroupNor, Norwegian subtitles Condition; sexmale, male sex; log (FreqWD), word frequency; log(Grammar), grammar test results (log); FilmSubeng, English subtitles when watching an English movie; FilmSubnor, Norwegian subtitles when watching an English movie; Cartoon, amount of time spent watching animated cartoons in English; Read_Norw, self-estimated Norwegian reading skills; Read_Eng, self-estimated English reading skills; Write_Eng, self-estimated English writing skills; Listen_Eng, composite factor created by the ratio of self-estimated English listening skills compared to self-estimated English speaking skills; Age: GroupEng, interaction of age and viewing condition with English subtitles; Age: GroupNor, interaction of age and viewing condition with Norwegian subtitles. Significance codes: (^*^) p ≤ 0.1; ^*^ p ≤ 0.05; ^**^ p ≤ 0.01.

**Table 7:** **Results of the inferential analysis of the word definition task (17-year-old group)**.

	**Estimate**	***SE***	***z*-value**	**Pr(>|z|)**
(Intercept)	−17.16533	7.04609	−2.436	0.01484^*^
log(Grammar)	5.02720	1.82148	2.760	0.00578^**^
log(FreqWD)	−0.57120	0.30673	−1.862	0.06257 (*)
Listen	0.44393	0.26054	1.704	0.08840 (*)
Eng_game	0.26633	0.06215	4.285	1.83e-05^***^

log(grammar), grammar test results (log); log(FreqWD), frequency of the words (log); Listen, composite factor created by the ratio of self-estimated English listening skills compared to self-estimated English speaking skills; Eng_game, amount of time spent playing English computer games. Significance codes: (^*^) p ≤ 0.1; ^*^ p ≤ 0.05; ^**^ p ≤ 0.01; ^***^ p ≤ 0.001.

**Table 8:** **Results of the inferential analysis of the word definition task (16-year-old group)**.

	**Estimate**	***SE***	***z*-value**	**Pr(>|z|)**
(Intercept)	−10.12281	1.49018	−6.793	1.10e-11^***^
GroupEng	−0.12572	0.17645	−0.713	0.4762
GroupNor	0.28114	0.20975	1.340	0.1801
log(frequencyWD)	−0.13184	0.09394	−1.403	0.1605
log(vocabulary)	1.08642	0.14594	7.444	9.74e-14^***^
GroupEng:log(frequencyWD)	−0.01348	0.05028	−0.268	0.7886
GroupNor:log(frequencyWD)	0.11875	0.05847	2.031	0.0422^*^

GroupEng, English subtitles Condition; GroupNor, Norwegian subtitles Condition; log(frequencyWD), frequency of the words (log); log(vocabulary), composite factor created by the ratio of the vocabulary test score compared to self-estimated English written skills (log); GroupEng:log(frequencyWD), word frequency with results from GroupEng; GroupNor:log(frequencyWD), word frequency with results from GroupNor. Significance codes: ^*^ p ≤ 0.05; ^***^ p ≤ 0.001.

The presence of subtitles did not predict performance of the 17-year-old participants on the word definition task. The most significant factor was, as in the comprehension questionnaire, the (self-reported) amount of time spent playing English computer games (*p* < 0.001). Also, the grammar test results predicted the participants' performance (*p* < 0.01). The self-estimated speaking skills of the participants were also marginally significant (*p* < 0.1). Finally, quite surprisingly, the analysis marginally indicated that the participants tended to find more frequent words more challenging to define (*p* < 0.1). This result is difficult to interpret, however, a clue might be that more frequent words are typically encountered in a wider variety of contexts, and, thus, more difficult to define semantically.

Subtitles did not predict the 16-year-old group performance on the word definition task either. The reason why the English and the Norwegian subtitles group still appear in this table is due to the interaction between subtitles Condition and word frequency in this task. Frequency alone is not a significant predictor of the results across Conditions. The only effect in this respect is for participants in the Norwegian subtitle Condition, suggesting that this group performed better when the frequency of the word was higher (*p* < 0.05). The most significant factor, however, was a composite one created from the ratio of the scores on the vocabulary test and the participants' self-estimated English writing skills (*p* < 0.001). This factor, which most likely reflects vocabulary knowledge (including orthographic representations) best predicted performance on the word definition task.

#### Word recall task

Like in the word definition task, subtitles were not predictive of the participants' performance on the word recall task, suggesting again that there were no long term effects of the subtitles. Other predictors were, however, found. The results from the inferential analysis of the word recall task are showed in Table 9.

**Table 9:** **Results of the inferential analysis of the word recall task (both age groups)**.

	**Estimate**	***SE***	***z*-value**	**Pr(>|z|)**
(Interce pt)	0.311592	0.341998	0.911	0.36225
Cartoon	−0.079072	0.028127	−2.811	0.00493^**^
Read_Norw	0.029137	0.051416	0.567	0.57093
Listen_Eng	0.005233	0.067037	0.078	0.93778
Total_Eng	0.141125	0.078840	1.790	0.07345(^*^)
Write_EngT	−0.086192	0.036128	−2.386	0.01705^*^

Cartoon, amount of time spent watching animated cartoons in English; Read_Norw, self-estimated Norwegian reading skills; Listen_Eng, composite factor created by the ratio of self-estimated English listening skills compared to self-estimated English speaking skills; Write_EngT, how often the participants write English text. Significance codes: (^*^) p ≤ 0.1; ^*^ p ≤ 0.05; ^**^ p ≤ 0.01.

Table 9 shows that the participants who had spent more time watching English cartoons performed better on the word recall task (*p* < 0.01). The time they had spent writing English was also predictive of the performance (*p* < 0.05), indicating the significance of writing and orthographic representations for performance on this task.

## Discussion

### Short term effects

Both the 16-year-old and the 17-year-old participants who watched the *Family Guy* episode with subtitles in either their L1 or their L2 performed better than the control groups on the comprehension questionnaire. This result emerged both in the combined and in the separate age-group analysis. Thus, positive short term effects of subtitles as a source of input and as a source facilitating processing of the authentic auditory input were found. Furthermore, for the 17-year-old group, the language of the subtitles did not seem to matter with both subtitling conditions having a similar effect (*p* < 0.01). Similar positive effects were found for the 16-year-old group, but, surprisingly, for that age group, the English subtitles were more facilitatory (*p* < 0.01) than the Norwegian subtitles (*p* < 0.05). These findings are in contrast to what we had expected, but support the views of Baltova (1999) who argues that the combination of auditory material in the target language (L2), verbal visual information, and nonverbal visual information in audio-visual material creates a better environment for learning than when only two of the three are available as input channels. When exposed to an animated cartoon in the L2, the students performed significantly better on comprehension of the contents of the episode when subtitles were available. Furthermore, we find evidence that target language subtitles enhance speech segmentation by providing the learner with orthographic information, in addition to the phonological one, and thus enhance the processing of the target language content (Mitterer and McQueen, 2009).

The combined age-group analysis revealed an important long-term effect of watching subtitled video material. This is reflected in the negative impact the habit of watching inter-lingual (native language) subtitles has on comprehension, as reported in the background questionnaire, and as seen in the predictive significance of this factor in the combined group analysis. A similar negative effect of frequent watching subtitled video with native language subtitles was also found in the combined analysis for the word definition task.

The results from both participant groups thus confirmed the overall initial hypothesis that having subtitles available would enhance the participants' performance, at least on the comprehension questionnaire. However, as it was hypothesized that the more advanced group would benefit more from authentic L2 subtitles as a result of higher proficiency, the lack of difference between the two subtitling Conditions in that group was surprising. Also, for the 16-year-old group it was assumed that native language (L1) subtitles would be more facilitative as a result of (somewhat) lower proficiency. However, the analysis revealed that the target language (L2) subtitles were more facilitative. One can argue that for the more advanced participants the language of the subtitles did not matter. What mattered was simply that they had access to a third input channel which assisted the comprehension of the contents of the audio-visual material.

The analysis of the 17-year-old group's performance can be argued to be contrary to the findings in Mitterer and McQueen (2009), Markham (1999), Vandergift (2011), and Vanderplank (1988), who found that L2 subtitles were more facilitatory. Mitterer and McQueen (2009) also argued that L1 subtitles would harm target language speech perception. However, our study indicates that native language subtitles may enhance comprehension of audio-visual material at least equally as target language subtitles. Minimally, these results argue against the view that the presence of native language subtitles harms the participants' perception of the auditory material. Bianchi and Ciabattoni (2008) found that target language subtitles were more facilitative for more advanced students, whereas native language subtitles were the better option for less advanced students. The results from our study, however, indicate that for more advanced students, the language of the subtitles is of a lesser importance, whereas for less advanced students, L2 subtitles were in fact more facilitatory. This is in line with Guillroy (1998), who argues that L2 subtitles can compensate for the challenging vocabulary in audio-visual material, and exactly this compensating effect of the English subtitles might have been a factor in boosting the performance of the 16-year-old participants in the English subtitles Condition. Moreover, we also found evidence that native language subtitles impact negatively on performance on comprehension from a long-term perspective, as revealed by the combined age-group analysis on both the comprehension task and the word definition task conducted 4 weeks after watching the test video.

An alternative explanation may be that the 16-year old participants in our study are advanced enough and are already at a high level of proficiency, e.g., compared to the participants in some of the studies reviewed here. This seems very likely in view of the advanced level of English competency in Norwegian school students overall (Alabau et al., 2002; Helland, 2008). Still, we find a difference between the age groups in the current study. We can interpret these results as consistent with earlier findings that target language subtitles are more facilitatory at high levels of proficiency, as reflected in the effects found in the 16-year-old group. It can then be speculated that the results of the 17-year-old group reflect a highly advanced proficiency level, where the language of the subtitles does not matter in skilled L2 comprehenders, especially on a single viewing instance (short-term). Furthermore, other factors, such as vocabulary size, grammar competence, daily L2 practices, such as watching target language subtitles and playing computer games are significant predictors of performance on the comprehension task, consistent with language learning research and the role of exposure to input (Mackey, 1999; Unsworth et al., 2014).

### Long term effects

In the combined analysis we found a long-term effect of the native language subtitle viewing Condition. However, this effect was modulated by age as seen in the interaction of age group and viewing condition. Furthermore, the separate analyses for each age group, revealed that subtitles were not predictive of performance on the word definition task or on the word recall task. As such, no long-term effects of the presence of subtitles on a single instance of viewing were found. Originally, we had expected to see an effect of the subtitles, and the tasks were designed directly based on the episode participants had watched 4 weeks earlier. One of the reasons for the lack of subtitling effects on these two tasks might be the long lapse between exposure to the stimuli and the testing. Four weeks is a long period of time and the episode was not watched again in this time period. Moreover, participants were exposed to the material only once. Indeed, many studies which fail to document implicit learning in the context of authentic input have been criticized for testing too late and only after single exposure to the material tested. Since the subtitles did have an effect on the short term comprehension task, it is unreasonable to assume that participants did not pay attention to the subtitles, and that the absence of long-term effects on a single exposure was not caused by lack of attention to the subtitles. Moreover, there is ample evidence that subtitles and auditory material are processed in parallel, with subtitles automatically read (d'Ydewalle and Van de Poel, 1999) and that participants attend visually to the text (Bisson et al., 2014). Conducting the long term effect tasks sooner after exposure to the stimuli might have potentially led to different results, though the notion of “long term” thus becomes open to debate. Alternatively, exposing the participants to subtitled audio-visual material regularly during the 4 week period might also have led to positive implicit learning outcomes.

We do find, however, long-term effects of subtitle viewing in the combined age-group analysis based on self-reported daily practice of watching English films with either native language or target language subtitles. Our results indicate that a preference for watching native language subtitles has a negative impact on comprehension, as well as lexical skills, such as assessing word definitions, and thus, indirectly confirms the importance of target language subtitles (as compared to no subtitles at all). Finally, the word recall task only taps (most probably, conscious) memory of having encountered the target word in the context of the video, and as such, the results from this task are limited to interpretation. As noted by Vandergift (2011), the long term effects of subtitles are unclear, and we suggest that further research should be conducted in this area.

### Other factors

In this study we found mainly evidence of short term effects of the use of subtitles. However, other factors from the participants' linguistic background and L2 practices are worth discussing.

The combined analysis for the word definition task and the word recall task revealed a strong effect of overall self-assessed English writing skills, as well as English grammar competence on the word definition task only, as measured on the Grammar pre-test. Both of these results suggest that overall L2 language competence, and specifically writing skills, impact on performance on a variety of lexical tasks and appear to underlie an important aspect of L2 lexical skills, most probably related to orthography and entrenching associations between form and meaning (Brown and Hulme, 1996).

For the 17-year-old group, the large effect of the amount of time spent playing English computer games (*p* < 0.001) on both the comprehension questionnaire and the word definition task is particularly interesting. Like films, computer games can be argued to provide authentic audio-visual material and to have an impact on the L2 acquisition of the learner. Computer games have similar visual features: they are animated and often contain both orthographic (textual) and auditory information. Thus students who are skilled players, are accustomed to interpreting meaning from animated L2 material, and might thus have an advantage on performance on comprehension in the cartoon task as well. Interacting with computer games in the L2 also requires learning new words in order to understand and proceed in the game, something which might have developed the player's heuristics skills as well as increased their vocabulary size. Indeed, there is a growing body of evidence suggesting incidental L2 learning in the context of computer games (Uzun et al., 2013; Sundqvist and Sylvén, 2014). The role of computer games can be accounted for within a situated and embodied cognition model of processing. On such a model, text comprehension is based on creating situation models that match the verbal content of the utterances (Kintsch, 1998; Zwaan, 1999). Computer games as a single factor influencing both participants' performance on the word definition task, and the comprehension task, is thus not so difficult to explain. Also, the effect of this factor can be mediated by (better) attentional skills, which can be tested in future work.

It is worthy to mention in the results for the 17-year-old group the predictive role of grammar competence on both comprehension and lexical skills (word definitions) (*p* < 0.05). One might argue that this is evidence that underlying grammar competence is an overall comprehensive predictor of performance on tasks in the L2, irrespective of their nature.

For the 16-year-old group, the results from the initial vocabulary test in the Norwegian subtitles Condition can be argued to be particularly interesting. In that group, vocabulary scores were an important factor both for comprehension (*p* < 0.001) and word knowledge (the word definition task) (*p* < 0.001), though for the latter it was a composite factor compared with self-estimated English writing skills. Indeed vocabulary knowledge has previously been established as a determinant of comprehension, particularly in the second language, though in younger participants (Lervåg and Aukrust, 2010). As the vocabulary test was taken before exposure to the material, we cannot view it as indicative of learning in this study. Instead, the results suggest that participants with better vocabulary knowledge (based on our testing) perform better on the comprehension test exactly as a result of a larger lexicon in the L2. The analysis of the result from the word definition task showed that the participants in the Norwegian subtitles Condition benefited less from the presence of subtitles than participants in the English subtitles Condition. A potential explanation can be sought in the Norwegian subtitles group's high scores on the vocabulary test, as this was generally more predictive of the results overall, thus highlighting the role of multiple factors in language acquisition. Furthermore, the analysis of the results from the comprehension task implies that participants in that group in all likelihood paid attention to the subtitles, still vocabulary knowledge superseded the importance of subtitles, suggesting that already obtained knowledge replaced the dependence on subtitles for higher competence learners.

We found that participants' self-assessed oral target language skills predict performance on the comprehension task. This result highlights the links between auditory perception skills in language learning and comprehension and indirectly support the idea that target language subtitles might be aiding in the process of L2 speech segmentation.

Worth mentioning is the contrast between results based on data from the self-reported L2 skills and L2-related activities. We found that viewing English video material (cartoons and films) impacts negatively on performance on the comprehension task (combined age-group analysis). This is surprising given that exposure to target language input should matter and rather have a positive effect. This is also in contrast to the negative long-term effect of viewing native (not-target) language subtitles. However, watching can be a passive activity, and it is unclear whether participants attend to the audio (language) of the video or rather to the visual features. It has been suggested that interaction is more important than passive contexts, and especially L2 contexts require active interaction (Mackey, 1999; Oliver and Mackey, 2003). Moreover, we find an effect of playing computer games, which are interactive and involve motivation for moving on to a next level of the game, confirming the idea that language learning is best situated in interactive contexts. This is also consistent with findings in L1 acquisition research.

## Conclusion

The question of whether second language learners should be trained using adapted materials or authentic materials is subject to debate. Many authors argue that learners should not be “protected” by adapted materials, and further suggest that authentic materials provide meaningful exposure to the target language (see Tomlinson, 2012 for a discussion). This study aimed to investigate the effects of subtitles in the context of authentic material (a cartoon video) on second language comprehension and potentially, second language acquisition for Norwegian learners of English. One hundred and fourteen participants in all participated in the study: 49 17-year-old students and 65 16-year-old students, who were all native speakers of Norwegian learning English as an L2 in high school. Both age groups were divided into three Conditions, where one group watched an episode of the American animated cartoon *Family Guy* with Norwegian subtitles, one group watched the episode with English subtitles, and one group watched the episode with no subtitles. On a comprehension questionnaire conducted immediately after watching the episode positive short term effects of both native language (L1) and target language (L2) subtitles were found for both age groups. However, no differences in terms of the language of the subtitles were found in the older and more advanced group. Four weeks later the participants responded to a word definition task and a word recall task to investigate potential long term effects of the subtitles. The only long-term effect of viewing subtitles on a single instance was found in the word definition task and was modulated by age. We found, however, that native language subtitles impact negatively on performance on the comprehension task. The results from this study suggest that the mere presence of subtitles as an additional source of information enhances learners' comprehension of the plot and content in animated audio-visual material in their L2. Since no major differences in terms of the language of the subtitles were found in the more advanced group, we argue that both intralanguage and interlanguage subtitles can aid target language comprehension in very advanced learners, most probably suggesting better consolidated vocabulary knowledge in that group. Furthermore, we found a difference between the two age groups in what best predicted performance on the two lexical tasks. While in the less proficient and younger group vocabulary status best predicted performance on both tasks (vocabulary predicts vocabulary), for the very advanced and older group, grammar was a stronger predictor, highlighting the importance of generic language competence and skills in L2 tasks for highly proficient L2 users. We also found an effect of written L2 skills on performance on both lexical tasks indicative of the role of orthography in vocabulary consolidation.

The current study has its limitations. We did not test partipants' working memory skills or other cognitive competencies known to affect language acquisition and use (Vulchanova et al., 2014). We did not test attentional skills, which have been shown to have a bidirectional relationship with language competence and skills (as seen in studies of a bilingualism, Bak et al., 2014). Other behavioral measures, such as reaction times or eye-gaze data when viewing the experimental video could have added to assessing participants' performance. These are objectives for future research.

### Conflict of interest statement

The authors declare that the research was conducted in the absence of any commercial or financial relationships that could be construed as a potential conflict of interest.
