# Supplementary material for: As naturalistic as it gets: subtitles in the English classroom in Norway
Source: Front Psychol. 2015 Jan 9;5:1510. doi: 10.3389/fpsyg.2014.01510 (PMC4288378; doi:10.3389/fpsyg.2014.01510)
Supplement: Supplementary file 1 [file DataSheet1.DOCX]

Appendix 1 – Comprehension questionnaire

*Note:* X= correct response

| \| **Deltakerkode:**  **(Fås av læreren)** \| \| --- \| |
| --- | --- |

**Select the correct alternative. Select only one alternative for each question.**

1. Jillian’s boyfriend is...

a. Stewie □

b. Peter □

c. Brian X □

d. Carl □

2. Stewie is...

a. A dog □

b. A girl □

c. A boy X □

d. A cat □

3. Brian is...

a. A dog X □

b. A girl □

c. A boy □

d. A cat □

4. “Pe-ople” is...

a. A television show □

b. A magazine X □

c. A movie □

d. A girl □

5. Peter and Jillian go to see...

a. Les Miserables □

b. Walt On Ice □

c. Disney On Snow □

d. Disney On Ice X □

6. The book Brian is writing is called...

a. Faster than the speed of love X □

b. Faster than the speed of lightning □

c. Fast and furious □

d. Faster than the speed of ice skaters                                  □

7. Lois...

a. Is in love with Brian                                                         □

b. Likes Brian’s book idea                                                    □

c. Wants to direct a movie based on Brian’s book □

d. Makes fun of Brian’s book idea X □

8. Stewie is looking for...

a. French fries                                                                       □

b. Fanta lemon                                                                      □

c. Graham crackers X □

d. Chocolate cake □

9. Jillian’s neighbour is...

a. A sailor                                                                              □

b. A pirate X                                                     □

c. An opera singer                                                                 □

d. A thief                                                                              □

10. Jillian ends the relationship because...

a. Her boyfriend is too lazy                                                   □

b. Her boyfriend lied about wanting to live with her X □

c. Her boyfriend wants to move to Africa                           □

d. Her boyfriend kissed another girl □

11. Stewie has...

a. An Australian accent                                                        □

b. An Indian accent                                                               □

c. An American accent                                                          □

d. A British accent X □

12. Meg goes to buy...

a. Diapers X □

b. Deodorant □

c. Underwear □

d. Magazines □

13. Carl is...

a. A hair-dresser                                                                    □

b. A shop manager X □

c. A sports commentator □

d. A movie star □

14. Chris is Meg's...

a. Baby □

b. Boyfriend □

c. Bingo partner □

d. Brother X □

15. Chris tries to pay for comic books with his...

a. Money □

b. Insects □

c. Pooh X □

d. Pee □

16. Carl and Chris discuss...

a. TV shows                                                                          □

b. Christmas                                                                          □

c. Cover girls                                                                         □

d. Movies X                                                                           □

17. Meg eventually gets a job for...

a. A television show □

b. A phone-sex line X □

c. A telemarketing company □

d. A travelling circus □

18. Chris helps Meg by...

a. Getting her job back X □

b. Saving her life □

c. Buying her jewelry □

d. Lending her money □
