# Supplementary material for: As naturalistic as it gets: subtitles in the English classroom in Norway
Source: Front Psychol. 2015 Jan 9;5:1510. doi: 10.3389/fpsyg.2014.01510 (PMC4288378; doi:10.3389/fpsyg.2014.01510)
Supplement: Supplementary file 2 [file DataSheet2.DOCX]

Appendix 2 – Word definition task

*Note:* X = correct response

| \| Deltakerkode: \| \| --- \| |
| --- | --- |

**Select the most appropriate definition for each word. Select only one alternative for each word.**

**1. A novel**
a. A short piece of narrative prose fiction
b. A fictional prose narrative of considerable length X
c. A prose narrative consisting of minimum two volumes
d. An encyclopedia

**2. Rent**
a. Money used to pay back a bank loan
b. Payment for living in an apartment/house which belongs to someone else X
c. An object free from dirt, stain, or impurities
d. Fixed compensation for services, paid to a person on a regular basis

**3. To hire**
a. To get rid of an employee
b. To tie a metallic rod
c. To employ someone X
d. To advance to a higher position

**4. A supervisor**
a. Someone who works in a supermarket
b. An employee
c. A person responsible for cleaning the bedroom
d. Someone in charge of a particular unit X

**5. To promote**
a. To raise someone to a higher rank X
b. To assign someone to a lower position
c. To give someone a second chance
d. To prefer someone to another

**6. A sequel**
a. A constitution
b. A continuation X
c. Someone who sneaks about
d. A conclusion

**7. To smother**
a. To cough
b. To bore
c. To die
d. To suffocate X

**8. A pamphlet**
a. A short piece of printed paper on a current topic X
b. A porcupine
c. A soft piece of toilet paper
d. A flying piece of printed paper on a current topic

**9. Off-putting**
a. Turning something off
b. Interesting
c. Cheating
d. Repelling X

**10. Hazy**
a. Hazardous
b. Undecided
c. Unclear X
d. Unsuited

**11. Disposable income**
a. The money someone pays in taxes
b. Diapers that can only be used once
c. The money someone has available to buy consumer goods X
d. The money someone has to borrow to afford to buy consumer goods

**12. A cruller**
a. Sweet cake dough fried in deep fat X
b. French fries
c. A fruit-flavored and glazed twisted candy roll
d. A hair curler

**13. Crabby**
a. Humorous
b. Ill-tempered X
c. Lazy
d. Annoying

**14. The gist of something**
a. A central idea X
b. A protagonist
c. A conception
d. A poison

**15. Braces**
a. A dental regulating tooth brush
b. An appliance that creates dental irregularities
c. A dental device that is used for flossing
d. An appliance that corrects dental irregularities X

**16. An underrated actress**
a. A female with low self-esteem
b. A female whose acting talent is underestimated X
c. An underestimated female magician’s assistant
d. A female with great acting talent who has low self-esteem

**17. A captive**
a. A person who only writes in capital letters
b. A person who has committed a crime
c. A person who is held against his will X
d. A person who is held responsible for an accident

**18. To stack**
a. To arrange things in a pile X
b. To organize things in a line
c. To hit someone with a stick
d. To arrange piles of hay in a line

**19. “No way”**
a. “You are joking”
b. “Not possibly” X
c. “Not a road”
d. “Never mind”

**20. To alphabetize**
a. To learn the alphabet
b. To arrange in alphabetical order X
c. To mark with letters
d. To organize by date

**21. Paper route**
a. The job of delivering newspapers regularly X
b. The road taken by the toilet paper after flushing
c. The job of delivering mail regularly
d. The process in which a newspaper is made

**22. Committed**
a. To be happy about a change
b. To leave someone out
c. To be bound to something X
d. To evolve into something more serious

**Idioms**

**23. To be game**
a. To be happy
b. To be agreeable to participate in something X
c. To be excited about an upcoming event
d. To be willing to play a game

**24. To take charge**
a. To be responsible
b. To reload batteries
c. To take control over something X
d. To make someone pay for something

**25. To make up one's mind**
a. To conclude a chapter in a book
b. To choose the direction of a play
c. To paint one’s face
d. To come to a decision X

**26. A pain in the ass**
a. A suppository pill
b. Something causing trouble X
c. A donkey with problems
d. Constipation

**27. To cheer up**
a. To buy new furniture
b. To view something from above
c. To become happy X
d. To sing cheerfully

**28. To get pushed around**
a. To do something bad
b. To be supported by someone
c. To be physically harassed
d. To do everything you are being told X

**29. To have a broken heart**
a. To experience great emotional pain X
b. To have a cardiac arrest
c. To feel a strong pain in the chest
d. To be broke

**30. Not my cup of tea**
a. A borrowed cup used for drinking tea
b. A type of tea you do not like
c. Something you enjoy or like
d. Something you do not like X
