# Supplementary material for: As naturalistic as it gets: subtitles in the English classroom in Norway
Source: Front Psychol. 2015 Jan 9;5:1510. doi: 10.3389/fpsyg.2014.01510 (PMC4288378; doi:10.3389/fpsyg.2014.01510)
Supplement: Supplementary file 3 [file DataSheet3.DOCX]

Appendix 3 – Word Recall Task

*Note:* o-occurred, n-did not occur, p-primed word, v-visually primed word

□ Pilot o

□ Hot o

□ Pirate v

□ Pressed p

□ Flat n

□ Equal o

□ Overpaid o

□ Error n

□ Job o

□ Dumb n

□ Ache n

□ Will do p

□ Work o

□ Bedroom v

□ Backpack p

□ Daughter n

□ Fatherly o

□ Kill n

□ Genuine p

□ Move in o

□ Basketball n

□ Film o

□ Married n

□ Sabbatical o

□ Shop v

□ Cry n

□ Cobbler o

□ Begin p

□ Aerial o

□ Immigrant o

□ Stunning n

□ Money o

□ Accuse n

□ Stock room o

□ This is functioning p

□ Biscuit p

□ Ugly n

□ (Be) sorry o

□ Pig p

□ Paycheck o

□ Cupboard n

□ Sexy p

□ Skating duet o

□ Dog v

□ Constitution n

□ Awesome o

□ State n

□ Brand-new o

□ Motherly n

□ Lonesome n

□ Paper towel o

□ Chick o

□ Dreadful p
