# Supplementary material for: As naturalistic as it gets: subtitles in the English classroom in Norway
Source: Front Psychol. 2015 Jan 9;5:1510. doi: 10.3389/fpsyg.2014.01510 (PMC4288378; doi:10.3389/fpsyg.2014.01510)
Supplement: Supplementary file 4 [file DataSheet4.DOCX]

## Appendix 4: Background questionnaire

**Bakgrunnsinformasjon for forskningsprosjekt om andrespråkforståelse**

Tusen takk for at du har sagt ja til å delta i vårt forskningsprosjekt om andrespråkforståelse. I dette skjemaet ber vi om bakgrunnsinformasjon som er nødvendig for at resultatene fra undersøkelsen skal kunne brukes.

Alle opplysningene du gir her, vil senere bli behandlet uten direkte gjenkjennende opplysninger. En kode knytter deg til dine opplysninger gjennom en deltakerliste. Det er kun autorisert personell knyttet til prosjektet som har adgang til deltakerlisten og som kan finne tilbake til infoen. Del B, C og D av dette skjemaet vil bare oppbevares med koden. All informasjon vil bli anonymisert ved prosjektslutt. Det vil ikke være mulig å identifisere deg i resultatene av studien når disse publiseres.
Legg merke til at skjemaet har 6 sider.

Med takknemlig hilsen,

Lisa Aurstad / Ingrid Kvitnes
Studenter ved lektorutdanning med master i språk, NTNU

**Part A: Personal information**

**Line of study and year: ____________________________________________________**

**Year of birth: ­­­­­­­­­­­­­­_________________________**

**Sex              □ Female                    □ Male**

**Municipality/city: ­­­­­­­­­­­­­­­­­­­__________________________________________**

| \| Participant code: \| \| --- \| |
| --- | --- |

**Part: Linguistic background**

**First language**

**Is Norwegian your first language?**
           □ Yes   □ No

**If yes, do you have any other first languages?**
        □ Yes   □ No

If yes, which language(s)? __________________________________________­­­­­­______

**Which language do you use at home?** ___________________________________________

**How often do you read texts in Norwegian?**
□ Every day     □ Several times every week   □ One or two times every week     □ Now and then      □ Never     

**How often do you write texts in Norwegian?**
□ Every day     □ Several times every week   □ One or two times every week     □ Now and then      □ Never

**English and other languages**

**How would you rate your English competence in each of the following areas?**

|  | **Basic** | **Intermediate** | **Advanced** | **Fluent** |
| --- | --- | --- | --- | --- |
| Reading |  |  |  |  |
| Writing |  |  |  |  |
| Speaking |  |  |  |  |
| Listening |  |  |  |  |
| In total |  |  |  |  |

**Have you lived in, or stayed in for a longer period of time, a country where English is the main language?**          □ Yes   □ No

If yes, how long did your stay(s) last?­­­­­­­____________________________

**Have you travelled to a country where English is the main language for a shorter period of time (less than 14 days)?**          □ Yes  □ No

**Have you lived in, or stayed in for a longer period of time, a country where English is not the main language?**          □ Yes    □ No

If yes, how long did your stay(s) last? ______________________________________

_____________________________________________________________________

**Which languages, besides your first language and English, do you know?**

| **Language** | **Level** |  |  |  |
| --- | --- | --- | --- | --- |
|  | **Basic** | **Intermediate** | **Advanced** | **Fluent** |
| German |  |  |  |  |
| French |  |  |  |  |
| Spanish |  |  |  |  |
| *- add language* |  |  |  |  |
| *- add language* |  |  |  |  |
| *- add language* |  |  |  |  |

**How often do you read texts in English?**
□ Every day     □ Several times every week   □ One or two times every week     □ Now and then      □ Never     

**How often do you write texts in English?**
□ Every day     □ Several times every week   □ One or two times every week     □ Now and then      □ Never     

**How often do you listen to/hear English?**
□ Every day     □ Several times every week   □ One or two times every week     □ Now and then      □ Never     

**How often do you watch series/films in English?**
□ Every day     □ Several times every week   □ One or two times every week     □ Now and then      □ Never     

**When you watch films in English, which of these alteratives do you use the most?**
□ Norwegian subtitles (first language)    □ English subtitles      □ No subtitles

**How often do you watch animated cartoons (series/films)?**
□ Every day     □ Several times every week   □ One or two times every week     □ Now and then      □ Never     

**Have you watched the animated cartoon “Family Guy”?**

□ Yes    □ No

If yes, to which extent? ____________________________________________

**How often do you play computer games in English?**
□ Every day     □ Several times every week   □ One or two times every week     □ Now and then      □ Never     

Which type of games do you play? _________________________________________

For how many hours per day? ________________________________

**How much time do you spend watching TV every day?**

□ 7 hours or more □ 5-6 hours □ 3-4 hours □ 1-2 hours □ I don’t watch TV

**Part C: Other factors in language acquisition**

**Do you have, or have you had, problems with vision other than using normal glasses?**      □ Yes    □ No

**Do you have, or have you had, problems with hearing?**      □ Yes     □ No

**Do you have, or have you had, language difficulties of any kind? (specific language problems, dyslexia, learning difficulties etc.)?**      □ Yes    □ No

If yes, please specify: ______________________________

**Do you have, or have you had, any other diagnosis that might affect language acquisition? (ADHA, autism etc)?**

□ Yes  □ No

**Are you left handed?**
      □ Yes    □ No

**Part D: Vocabulary test and grammar test**

**Vocabulary test result:**

Round 1.

Round 2.

**Grammar test result:**
